# Preoperative prediction of the localization of a solitary fibrous tumor using four-dimensional computed tomography: a case report

**DOI:** 10.1093/jscr/rjab184

**Published:** 2021-06-05

**Authors:** Daisuke Nakamura, Nobutaka Kobayashi, Masahisa Miyazawa, Hidetoshi Satomi

**Affiliations:** Department of Thoracic Surgery, Japanese Red Cross Society Nagano Hospital, Nagano, Nagano, Japan; Department of Thoracic Surgery, Japanese Red Cross Society Nagano Hospital, Nagano, Nagano, Japan; Department of Thoracic Surgery, Japanese Red Cross Society Nagano Hospital, Nagano, Nagano, Japan; Department of Pathology, Japanese Red Cross Society Nagano Hospital, Nagano, Nagano, Japan

## Abstract

Solitary fibrous tumors of the pleura (SFTP) are relatively rare primary pleural tumors. Four-dimensional computed tomography (4D-CT) is reportedly useful in assessing parietal pleural invasion and adhesion in patients with lung cancer. We report a case in which 4D-CT was performed to evaluate SFTP localization and parietal pleural invasion and adhesions. A 62-year-old female presented with an abnormality on a chest radiograph. Chest CT revealed a well-demarcated solid nodule in the left lower lobe adjacent to the pleura. We considered that the tumor was intrapulmonary or arose from the visceral pleura, without adhesion or invasion to the chest wall based on 4D-CT. Primary lung cancer was suspected, and the tumor was resected. Pathological diagnosis revealed an SFTP. This case suggests that 4D-CT is useful in predicting the localization of SFTP and other thoracic tumors, assessing chest wall adhesion and invasion, and making surgical strategies.

## INTRODUCTION

Solitary fibrous tumors of the pleura (SFTP) are relatively rare, accounting for <5% of all pleural tumors [1]. Approximately 80% of SFTPs originate from the visceral pleura and the remaining 20% originate from the parietal pleura [2]. Four-dimensional computed tomography (4D-CT) has been reported to be useful in assessing parietal pleural invasion and adhesion in patients with peripheral lung cancer [3, 4]. However, there have been no reports, to our knowledge, of the use of 4D-CT in predicting the localization or assessing parietal pleural invasion and adhesion of SFTP. We report a case in which 4D-CT was performed to evaluate SFTP localization and adhesions.

## CASE REPORT

**Figure 1:**
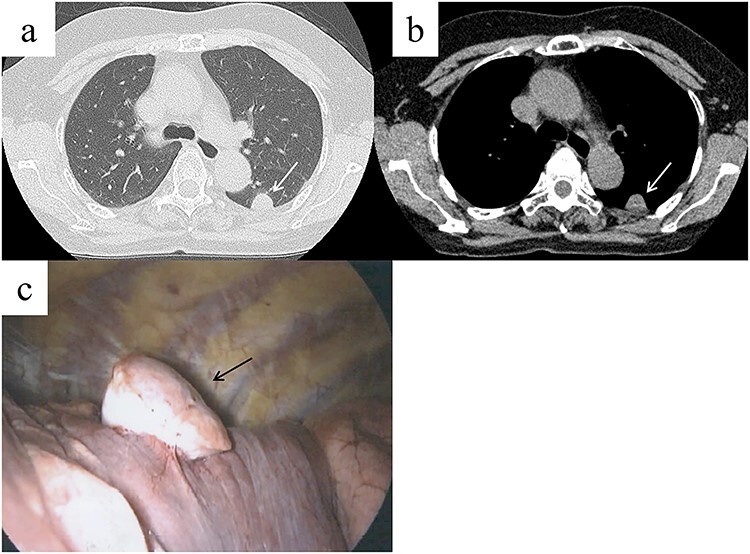
Chest computed tomography (CT) shows a well-demarcated solid nodule in the left lower lobe adjacent to the pleura (**a**, **b**, white arrow). Intraoperative findings show a stemmed tumor arising from the visceral pleura of the left lower lobe (**c**, black arrow).

A 62-year-old female visited our hospital because of an abnormality that was detected on a chest radiograph during a routine health screening. The patient was a nonsmoker and was asymptomatic. She had undergone surgery for papillary thyroid cancer 5 years prior. Physical examination and laboratory test results were normal. Chest CT revealed a 22-mm well-demarcated solid nodule in the lower lobe of the left lung, adjacent to the pleura (Fig. 1a and b). 4D-CT using a 320-slice multidetector CT scan was performed to visualize the tumor localization and to determine the necessary surgical approach after the patient provided informed consent. The CT was continuously active while the patient performed full inspiration and expiration. The 4D-CT revealed that the nodule moved differently from the parietal pleural during breathing (Video 1). The tumor was determined to be intrapulmonary, or potential visceral pleural origin, and without evidence of adhesion to or invasion of the chest wall. Primary lung cancer or another thoracic tumor was suspected, and surgical resection was required for the final diagnosis. Based on the 4D-CT, we considered that thoracoscopic lung resection was feasible and chest wall resection was not necessary.

**Figure 2:**
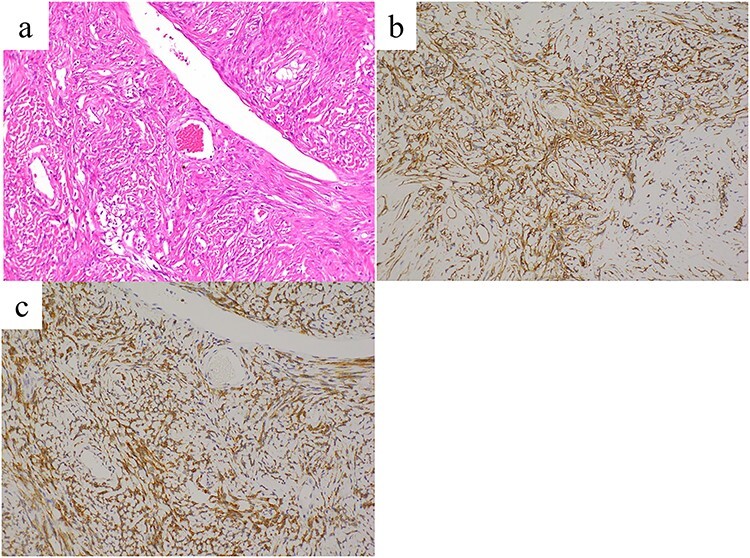
Histopathological findings reveal proliferating spindle cells with intermittent collagen fibers (**a**, hematoxylin and eosin staining). These tumor cells are immunohistochemically positive for (**b**) CD34 and (**c**) bcl-2. Images were taken at ×20 magnification.

A stemmed tumor arising from the visceral pleura of the left lower lobe was observed intraoperatively without adhesion to the parietal pleura (Fig. 1c). Wedge resection was performed using video-assisted thoracic surgery (VATS). The intraoperative pathological diagnosis of the tumor was SFTP. Microscopic examination of the resected specimen revealed spindle cells exhibiting a patternless proliferation with intermittent collagen fibers (Fig. 2a). Immunohistochemistry revealed that the tumor cells were positive for CD34 and bcl-2 (Fig. 2b and c), and this confirmed the diagnosis of SFTP. The patient’s postoperative course was uneventful and no signs of recurrence were observed at the 1-year follow-up. Informed consent was obtained from the patient for publication of this case report.

## DISCUSSION

The incidence of SFTP is 2.8 per 100 000 patients and is more frequent in people aged 50–70 years, without sex-specific differences [1]. SFTP is usually asymptomatic but has been associated with cough, chest pain, dyspnea and fever. In addition, hypoglycemia due to increased insulin-like growth factor-II production has also been reported [5]. Typically, SFTP presents as a solitary, localized mass with a smooth surface; however, it is difficult to diagnose using imaging. Most SFTPs are histologically benign, with proliferating spindle-shaped cells and collagen fibrils that are patternless [6]. Immunohistochemically, most SFTPs are positive for CD34, bcl2 and CD99 [7]. Complete surgical resection is the definitive treatment for SFTP and provides good long-term survival. However, even with benign tumors, local recurrence has been reported [8]. Rarely, delayed recurrence after >10 years postoperatively has been observed, highlighting the need for long-term follow-up [8].

4D-CT is a relatively new imaging technology, which was reported to be useful in assessing parietal pleural invasion and adhesion in peripheral lung cancer by observing tumor movement during breathing [3, 4]. However, to the best of our knowledge, preoperative prediction of tumor localization of SFTP and assessment of parietal pleural invasion and adhesions using 4D-CT have not been reported. In our case, preoperative 4D-CT revealed a tumor of visceral pleura or intrapulmonary origin because the tumor moved independently from the chest wall during breathing. Additionally, 4D-CT allowed visualization of adhesion and chest wall invasion.

The extrapleural sign is the gentle rise of a shadow from the chest wall on CT or X-ray when the lesion is outside the lung. This sign is useful in identifying the origin of a thoracic tumor. However, the location of an SFTP is reportedly difficult to predict [9]. Accurate evaluation of thoracic tumor localization and adhesion to the chest wall based on 4D-CT allows VATS to assess if surgery is possible, reduce the number of ports, and position ports appropriately. CT is routinely performed to visualize thoracic tumors; therefore, Choong *et al*. [10] reported that the addition of 4D-CT may be beneficial for accurate assessment of lung cancer invasion of the parietal pleura. 4D-CT can be performed simultaneously with conventional CT and could reduce the burden on the patient by eliminating the need for additional examinations. One challenge with using 4D-CT is that it requires relatively high radiation exposure compared to conventional CT. Sakuma *et al*. [11] reported that the total radiation exposure of 4D-CT ranged from 4.2 to 6.1 mSv. It may be possible to reduce the radiation dose by adjusting the scanning range of 4D-CT.

In conclusion, we report a case of a patient with SFTP located adjacent to the pleura in whom preoperative tumor assessment was performed using 4D-CT. This report demonstrates that 4D-CT is useful in predicting the localization of SFTP and also invasion of and adhesion to the chest wall. Because of its surgical advantages, 4D-CT should be actively used in evaluating thoracic tumors that are adjacent to the pleura.

## Supplementary Material

Video_1_rjab184
